# Angiotensin-converting enzyme 2 improves hepatic insulin resistance by regulating GABAergic signaling in the liver

**DOI:** 10.1016/j.jbc.2022.102603

**Published:** 2022-10-18

**Authors:** Qi Chen, Yuanyuan Gao, Fengying Yang, Hongjun Deng, Ying Wang, Li Yuan

**Affiliations:** Department of Endocrinology, Union Hospital, Tongji Medical College, Huazhong University of Science and Technology, Wuhan, China

**Keywords:** insulin resistance, angiotensin-converting enzyme 2, gamma-aminobutyric acid, hepatic steatosis, inflammation, A1–7, angiotensin 1–7, ACC, acetyl-CoA carboxylase, ACE2, angiotensin-converting enzyme 2, ACOX-1, acyl-CoA oxidase 1, AngI, angiotensin II, AT1R, type 1 angiotensin receptor, AVV, adeno-associated virus, CPT, carnitine palmityl transferase, CT, computed tomography, DMEM, Dulbecco's modified Eagle's medium, FAS, fatty acid synthase, GABA, gamma-aminobutyric acid, GAD, glutamate decarboxylase, HFD, high-fat diet, IL-1β, interleukin 1β, IR, insulin resistance, LV, lentivirus vector, NC, negative control, PA, palmitic acid, RAS, renin–angiotensin system, SD, standard diet, TC, total cholesterol, TG, triglyceride, TNF-α, tumor necrosis factor alpha

## Abstract

The angiotensin-converting enzyme 2 (ACE2)/angiotensin 1–7/MAS axis and the gamma-aminobutyric acid (GABA)ergic signaling system have both been shown to have the dual potential to improve insulin resistance (IR) and hepatic steatosis associated with obesity in the liver. Recent studies have demonstrated that ACE2 can regulate the GABA signal in various tissues. Notwithstanding this evidence, the functional relationship between ACE2 and GABA signal in the liver under IR remains elusive. Here, we used high-fat diet–induced models of IR in C57BL/6 mice as well as ACE2KO and adeno-associated virus-mediated ACE2 overexpression mouse models to address this knowledge gap. Our analysis showed that glutamate decarboxylase (GAD)67/GABA signaling was weakened in the liver during IR, whereas the expression of GAD67 and GABA decreased significantly in ACE2KO mice. Furthermore, exogenous administration of angiotensin 1–7 and adeno-associated virus- or lentivirus-mediated overexpression of ACE2 significantly increased hepatic GABA signaling in models of IR both *in vivo* and *in vitro*. We found that this treatment prevented lipid accumulation and promoted fatty acid β oxidation in hepatocytes as well as inhibited the expression of gluconeogenesis- and inflammation-related genes, which could be reversed by allylglycine, a specific GAD67 inhibitor. Collectively, our findings show that signaling via the ACE2/A1–7/MAS axis can improve hepatic IR by regulating hepatic GABA signaling. We propose that this research might indicate a potential strategy for the management of diabetes.

Type 2 diabetes is a chronic metabolic disease closely associated with a series of cardiovascular complications and increased mortality from related complications (1). Obese patients with type 2 diabetes are often accompanied by insulin resistance (IR). In the IR state, insulin signals in the target organs of the body are inhibited, which can induce abnormal glucose tolerance (2). The liver is one of the main target organs for insulin action and plays an essential role in the regulation of glucose and lipid metabolism (3). This study of liver IR can provide a new theoretical basis for the regulation and treatment of glucose and lipid metabolism disorders.

Gamma-aminobutyric acid (GABA) is an endogenous amino acid whose function depends on the GABA signal system. It is synthesized primarily by the decarboxylation of glutamate by glutamate decarboxylase (GAD65/67) and is capable of mediating a wide variety of physiological functions through its GABA receptor (4, 5). There is compelling evidence that islet β cells can produce and secret a large amount of GABA. On the one hand, it acts on beta cells to promote beta-cell proliferation and insulin secretion; on the other hand, it acts on alpha cells to inhibit glucagon secretion. The biphasic regulation of blood glucose by GABA has become a new target for diabetes treatment (6, 7, 8). In addition to direct blood glucose regulation, GABA is widely distributed in insulin-sensitive tissues (9, 10, 11). The liver is locally rich in GABAergic signaling. GABA administration can significantly alleviate inflammation and oxidative stress to hepatocytes, thereby reducing hepatocyte apoptosis in acute liver injury, indicating that GABA signal has a significant protective effect on the liver (12, 13). GABA can also restore insulin signal transduction by regulating hepatic lipid metabolism–related genes to improve IR (14, 15). However, only few studies are available on the factors that regulate the expression and activity of the GABAergic signal system in the liver.

The angiotensin-converting enzyme 2 (ACE2)/angiotensin 1–7 (A1–7)/MAS axis is a crucial component of the renin–angiotensin system (RAS) and can antagonize the classical ACE/angiotensin II (AngII)/type 1 angiotensin receptor (AT1R) axis (16, 17), exerting a positive regulatory role. A1–7 is a vital effector molecule of the ACE2/A1–7/MAS axis, produced by ACE2 degradation of AngII and inhibits most of the damaging effects of AngII on the body (18). An increase in the ratio of A1–7 to AngⅡ in serum can enhance the vascular endothelial function of patients with type 2 diabetes (19), reduce the inflammatory response of tissues and organs, and improve the prognosis of patients with diabetes (20, 21). An increasing volume of data shows that the RAS is also active in the liver. The use of AT1R inhibitors to block the ACE/AngII/AT1R axis or increase the ACE2/A1–7/MAS axis's activity can significantly enhance the hepatic tissue's sensitivity to insulin signals by inhibiting the infiltration of inflammatory factors and stress response, thereby can reduce hepatic IR (22, 23, 24). Our previous studies also demonstrated that the hepatic local RAS was overactivated in mice fed with a high-fat diet (HFD), and the upregulation of ACE2/A1–7/MAS axis activity by exogenous administration of A1–7 modified abnormal glucose and lipid metabolism in the liver (25). These results suggest that ACE2/A1–7/MAS axis may be an effective target for treating hepatic IR, but the specific mechanism remains unclear.

In recent years, studies have found that overexpression of ACE2 in the central nervous system can increase the release of GABA in the presynapse, affecting its neurotransmission and function to improve anxiety in mice (26, 27). Our previous studies have shown that ACE2/A1–7/MAS axis attenuates beta-cell function by regulating GAD67/GABA signaling in islets during metabolic stress (28). The aforementioned research confirms various interactions between the RAS and GABA signal. It is well established that both RAS and GABA signal regulate hepatic IR. However, it is unclear whether there is a functional correlation between the ACE2/A1–7/MAS axis and GABA signaling in the liver. Therefore, this study aims to explore the mutual relationship between the hepatic ACE2/A1–7/MAS axis and the GABAergic signaling system and the effect of this relationship in IR. Here, we demonstrate that ACE2/A1–7/MAS axis can alleviate hepatic IR by regulating the expression of the hepatic GABAergic signaling system in HFD-induced mice and insulin-resistant cells.

## Results

### Upregulation of the ACE2/A1–7/MAS axis and GABA reduces body weight and improves glucose intolerance in HFD mice

**Figure 1:**
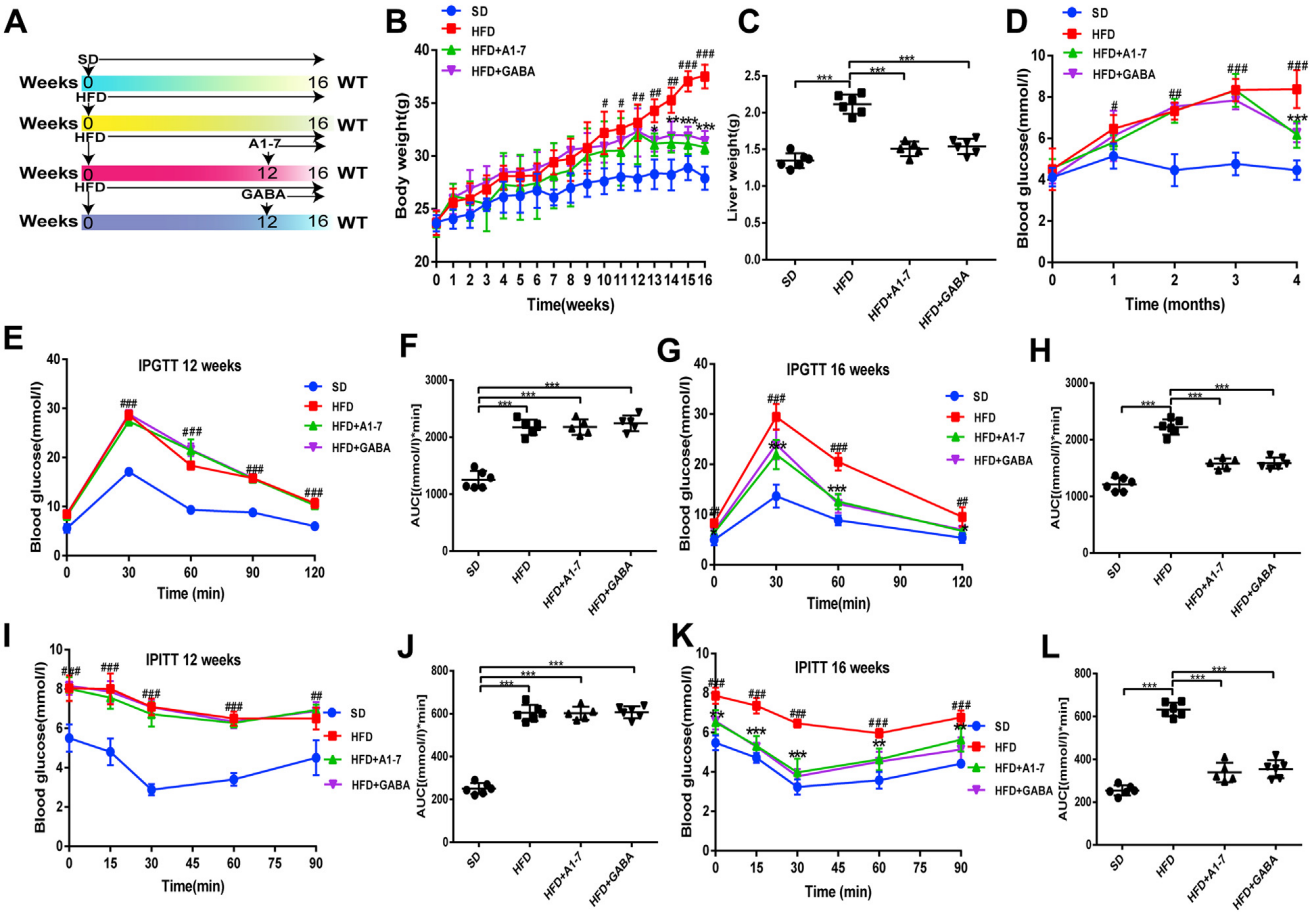
**A1–7 and GABA reduce body weight and improve glucose Intolerance in HFD-fed mice.***A*, experimental schedule. *B*, body weight. *C*, liver weight. *D*, fasting blood glucose levels. *E*–*H*, IPGTT was performed at 12 (*E*) and 16 weeks (*G*), which were fasted for 12 h, and the AUC of the IPGTT (*F* and *H*). *I*–*L*, IPITT was performed at 12 (*I*) and 16 weeks (*K*), which were fasted for 12 h, and the AUC of IPITT (*J* and *L*). Values were expressed as means ± SEM (n = 5 or n = 6). ^#^*p* < 0.05, ^##^*p* < 0.01, ^###^*p* < 0.001 *versus* SD group; ^∗^*p* < 0.05, ^∗∗^*p* < 0.01, ^∗∗∗^*p* < 0.001 *versus* HFD group for (*B*–*L*); A1–7, HFD mice received angiotensin 1 to 7 (576 μg/kg/day, i.p.); GABA, HFD mice received gamma-aminobutyric acid (6 mg/ml in drinking water). AUC, area under the curve; GABA, gamma-aminobutyric acid; HFD, high-fat diet; IPGTT, intraperitoneal glucose tolerance testing; IPITT, intraperitoneal insulin tolerance testing; SD, standard diet.

**Supplemental Figure S1:**
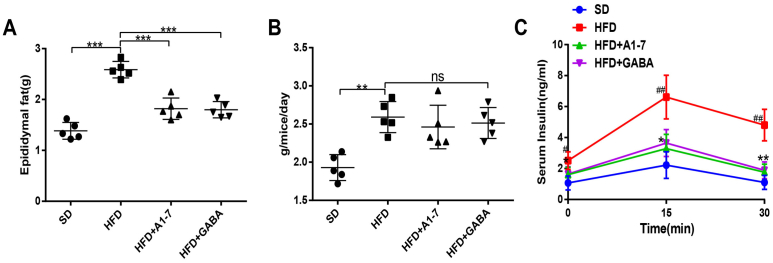


Previous studies suggest that ACE2/A1–7/MAS axis and GABA are downregulated in HFD mice (18, 28), indicating a regulatory role of the ACE2/A1–7/MAS axis and GABA in the development of IR. To confirm their beneficial effects in HFD-fed mice (Fig. 1, *A* and *B*), we first investigated the metabolic phenotypes of mice. HFD pretreatment results in a time-dependent manner in body weight gain during the modeling period. In addition, the liver and fat weights of the HFD group were higher than those of mice fed standard diet (SD). However, the administration of A1–7 or GABA prevented the increased parameters induced by HFD (Fig. 1*C* and S1*A*). Notably, A1–7 or GABA monotherapy did not affect food intake in HFD-fed mice (Fig. S1*B*). After feeding HFD for 16 weeks, the mice developed mild hyperglycemia, whereas fasting blood glucose levels decreased in the A1–7 or GABA groups (Fig. 1*D*). Consistently, at week 12, the area under the blood glucose curve of glucose tolerance test and insulin tolerance test for the HFD groups significantly increased compared with the SD group, which manifests a remarkable impairment of glucose tolerance and systemic insulin sensitivity in the HFD-fed mice (Fig. 1, *E–H*). A1–7 or GABA monotherapy reduced the area under the blood glucose curve of glucose tolerance test and insulin tolerance test, alleviated HFD-induced hyperinsulinemia, and thus improved glucose tolerance and insulin sensitivity (Figs. 1, *I–L* and S1*C*). Together, these results showed that treatment with ACE2/A1–7/MAS axis and GABA could normalize body weight and glucose and improve HFD-induced glucose tolerance.

### Upregulation of the ACE2/A1–7/MAS axis and GABA alleviates HFD-induced hepatic steatosis

**Figure 2:**
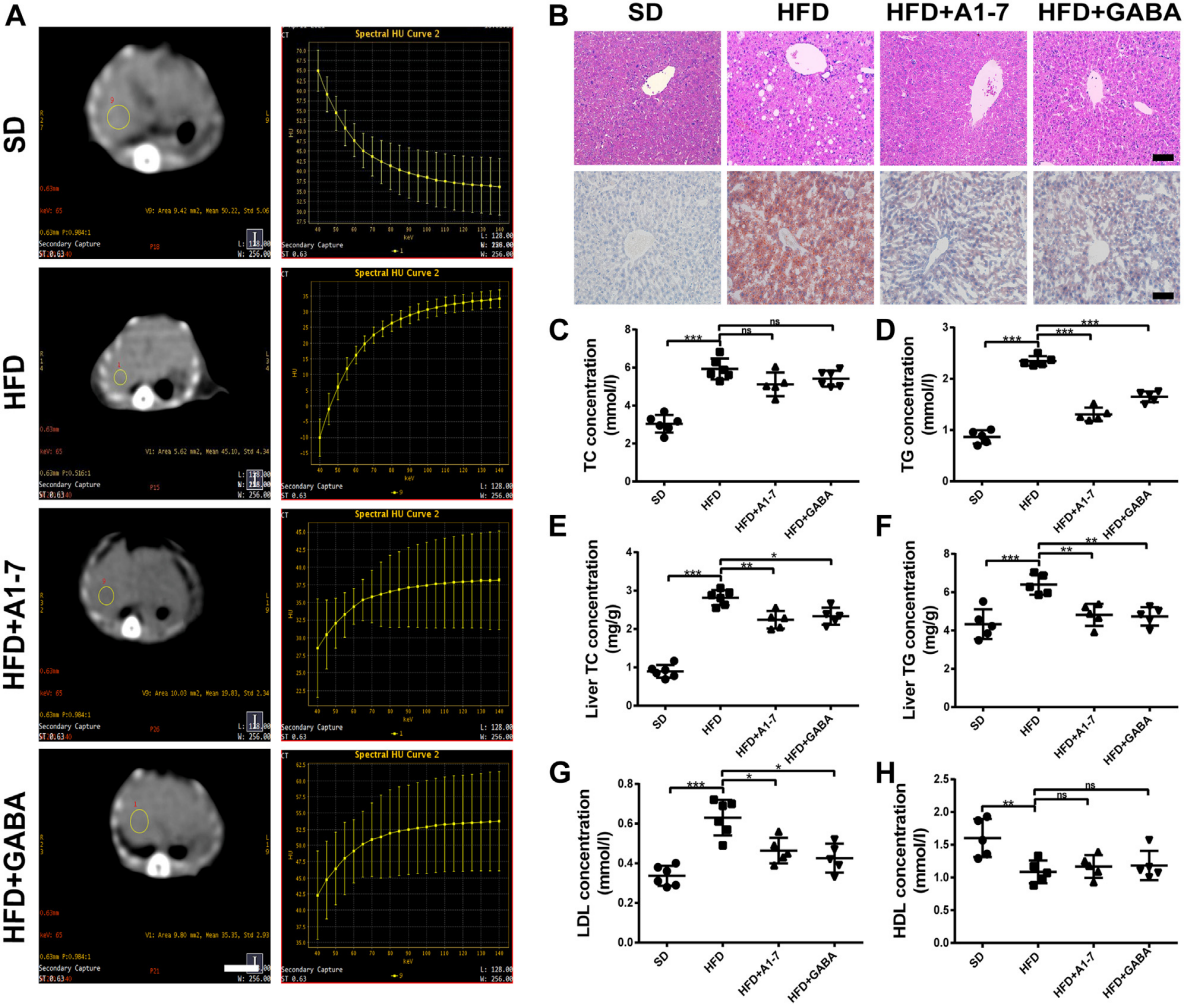
**Upregulation of A1–7 and GABA alleviates HFD-induced hepatic steatosis.***A*, representative 65 keV single-light CT image of the liver (*left*) and energy spectrum curve (*right*) of the HFD-fed mice. The scale bars represent 10 mm. *B*, liver tissue of H&E staining and Oil *Red* O staining. The scale bars represent 50 μm. *C* and *D*, serum levels of TC (*C*) and TG (*D*). *E* and *F*, the statistical charts of TC (*E*) and TG (*F*) content in liver tissues. *G* and *H*, serum levels of LDL (*G*) and HDL (*H*). Values were expressed as means ± SEM (n = 5 or n = 6). ^∗^*p* < 0.05, ^∗∗^*p* < 0.01, ^∗∗∗^*p* < 0.001. A1–7, HFD mice received angiotensin 1 to 7 (576 μg/kg/day, i.p.); CT, computed tomography; GABA, HFD mice received gamma-aminobutyric acid (6 mg/ml in drinking water); HDL, high-density lipoprotein; HFD, high-fat diet; LDL, low-density lipoprotein; ns, no statistical difference; SD, standard diet; TC, total cholesterol; TG, triglyceride.

We then explored ACE2/A1–7/MAS axis and GABA protective effect on hepatic steatosis. As demonstrated in Figure 2*A*, the energy spectrum curve of mouse liver computed tomography (CT) revealed that the HFD-fed mice exhibited marked fatty liver compared with the normal mice. However, after administration of A1–7 or GABA, the energy spectrum curve shifted downward, and the CT value of the liver was significantly increased, indicating a significant reduction in liver fat content. To directly assess whether A1–7 or GABA treatment influences liver morphology, liver tissue of the mice was detected *via* H&E staining and Oil Red O staining. As expected, histopathological analysis of H&E or Oil Red O-stained liver sections showed hepatocyte swelling and marked microvesicular steatosis in the HFD group (Fig. 2*B*). A notably alleviated accumulation of lipid deposition in the liver could be observed in the HFD + A–7 or GABA group (Fig. 2*B*). In parallel with the histological findings, the serum triglyceride (TG) and liver total cholesterol (TC) and TG levels were significantly ameliorated after A1–7 or GABA treatment compared with the HFD group, suggesting better liver lipid metabolic profile. TC levels showed no marked difference compared with the HFD group (Fig. 2, *C–F*). Moreover, the plasma serological LDL level significantly increased compared with the SD group, whereas HDL levels declined in the HFD group. A1–7 or GABA intervention resulted in a significant reduction in LDL level, whereas the decline in HDL level was not statistically different compared with the HFD group (Fig. 2, *G* and *H*).

### A1–7 and GABA treatment regulates glucolipid metabolism and inflammation-related gene expression in HFD mice

**Figure 3:**
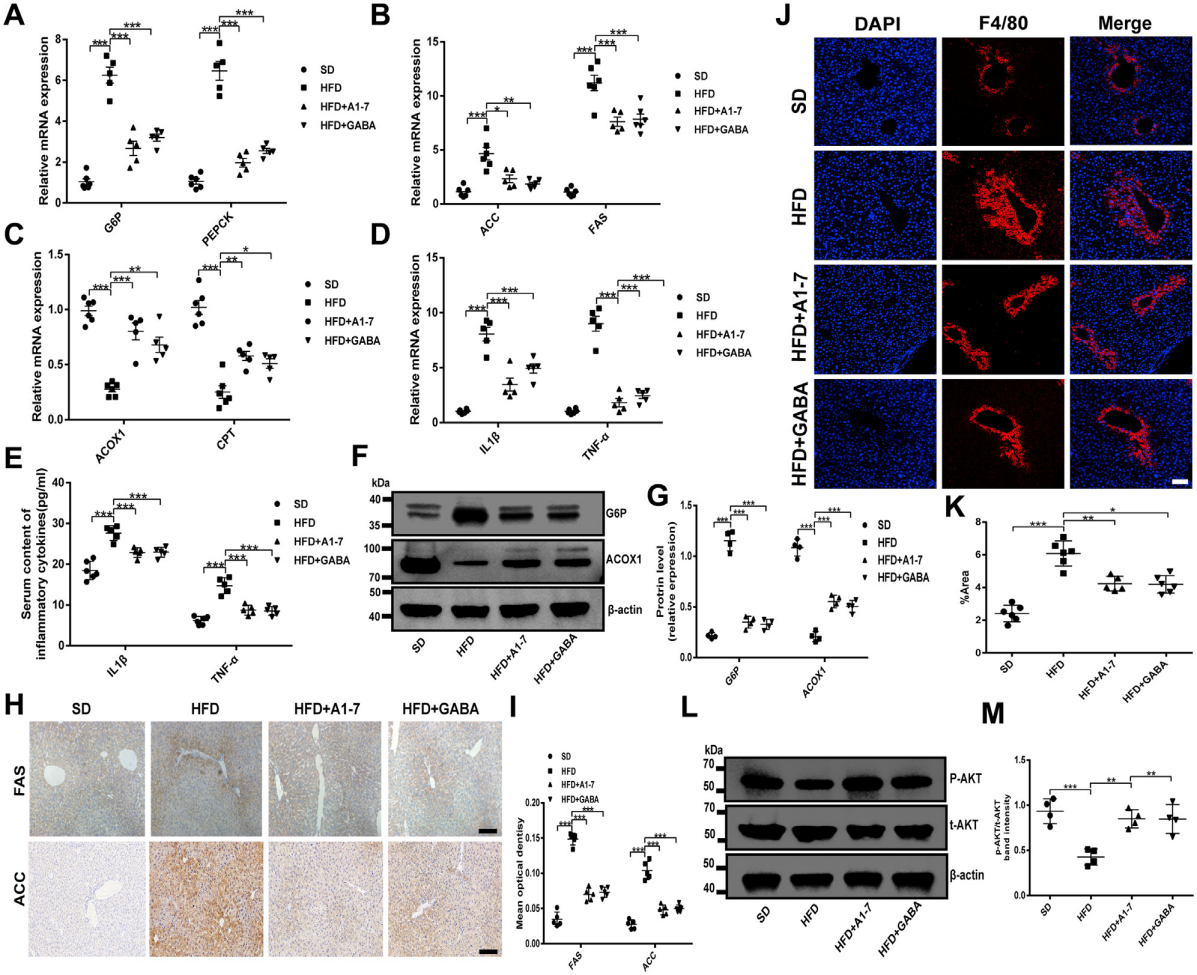
**A1–7 and GABA ameliorate hepatic steatosis by regulating glucolipid metabolism and inflammation in HFD-fed mice.***A*–*D*, RT–PCR was performed to analyze the liver mRNA levels of gluconeogenesis-related genes G6P and PEPCK (*A*), fatty acid synthesis–related genes ACC and FAS (*B*), fatty acid oxidation–related genes ACOX1 and CPT (*C*), and inflammation-related genes IL-1β and TNF-α (*D*). *E*, serum IL-1β and TNF-α concentration. *F* and *G*, the protein expression of G6P and ACOX1 in liver tissue. *H* and *I*, immunohistochemical staining was demonstrated to analyze FAS and ACC expression in liver tissues (*H*). The scatter diagram represents the mean intensity of FAS and ACC in the liver (*I*). The scale bars represent 50 μm. *J* and *K*, immunofluorescence staining was demonstrated to analyze F4/80 expression in liver tissue (*J*). The scale bars represent 50 μm. The scatter diagram shows the average absorbance of F4/80 in the liver (*K*). *L* and *M*, the protein expression of AKT phosphorylation in liver tissue and densitometric quantification. Values were expressed as means ± SEM (n = 5 or n = 6). ^∗^*p* < 0.05, ^∗∗^*p* < 0.01, and ^∗∗∗^*p* < 0.001. A1–7, HFD mice received angiotensin 1 to 7 (576 μg/kg/day, i.p.); ACC, acetyl-CoA carboxylase; ACOX1, acyl-CoA oxidase 1; CPT, carnitine palmityl transferase; FAS, fatty acid synthase; GABA, HFD mice received gamma-aminobutyric acid (6 mg/ml in drinking water); HFD, high-fat diet; IL-1β, interleukin 1β; SD, standard diet; TNF-α, tumor necrosis factor alpha.

To gain insights into the effects of the ACE2/A1–7/MAS axis and GABA on HFD-induced IR and related metabolic features, we explored the effects of A1–7 or GABA on the expression of genes involved in glycolipid metabolism and inflammation. A sign of hepatic IR is increased glucose production. Indeed, the expressions of PEPCK and G6Pase, which are key genes involved in the regulation of gluconeogenesis formation in the liver, were upregulated by HFD induced. However, A1–7 or GABA treatment significantly inhibited their overproduction (Fig. 3, *A*, *F* and *G*), as evidence of successful inhibition of endogenous glucose production. To help elucidate whether the reduction of hepatic TG and TC accumulation by A1–7 or GABA treatment was mediated by the suppression of lipogenesis, we assessed the gene expression levels of the fatty acid β oxidation genes (acyl-CoA oxidase 1 [ACOX-1] and carnitine palmityl transferase [CPT]) and fatty acid synthesis genes (acetyl-CoA carboxylase [ACC] and fatty acid synthase [FAS]). As shown in Figure 3, *B*, *C* and *F–I*, hepatic ACOX-1 and CPT expression decreased in HFD-fed mice, whereas the expression of ACC and FAS was significantly increased in the HFD group compared with the normal control mice. Nevertheless, the administration of A1–7 or GABA alone upregulated expression of the lipolysis-related genes (ACOX-1 and CPT) and inhibited the expression of ACC and FAS, which alleviated the lipid accumulation induced by HFD (Fig. 3, *B* and *C*, and *F–I*). Chronic low-grade inflammation leads to impaired insulin signaling, thereby promoting the progression of hepatic IR. As indicated in Figure 3*D*, the HFD promoted a dramatic inflammatory response, mainly manifested in an increase of interleukin 1β (IL-1β) and tumor necrosis factor alpha (TNF-α) expression in the livers compared with the SD group. Whereas treated with A1–7 or GABA significantly abated IL-1β and TNF-α levels in the livers of the HFD-fed mice. A significant increase in serum IL-1β and TNF-α levels were also observed in HFD-fed mice (Fig. 3*E*). As expected, treatment with A1–7 or GABA inhibited this increase (Fig. 3*E*). Prior studies showed that the polarization of macrophages in the liver affects the regular operation of liver function. In particular, excessive infiltration and proinflammatory activation of macrophages result in various diseases, including type 2 diabetes (29, 30). To further investigate the therapeutic effect of A1–7 or GABA on inflammation, the macrophage levels in liver tissue were detected by immunofluorescence. In agreement, immunofluorescence staining of F4/80 showed a higher abundance of macrophages in the liver tissue of HFD-fed mice, which was memorably suppressed by A1–7 or GABA treatment (Fig. 3, *J* and *K*). PI3K/AKT signaling pathways are a canonical pathway downstream of insulin. As expected, ACE2 and GABA intervention significantly upregulated liver phosphorylated AKT levels in HFD-fed mice (Fig. 3, *L* and *M*). As a whole, these results suggest that ACE2/A1–7/MAS axis and GABA regulate glucolipid metabolism and attenuate inflammatory response in HFD-fed mice.

### The ACE2/A1–7/MAS axis regulates hepatic GABAergic signaling expression in HFD mice

**Figure 4:**
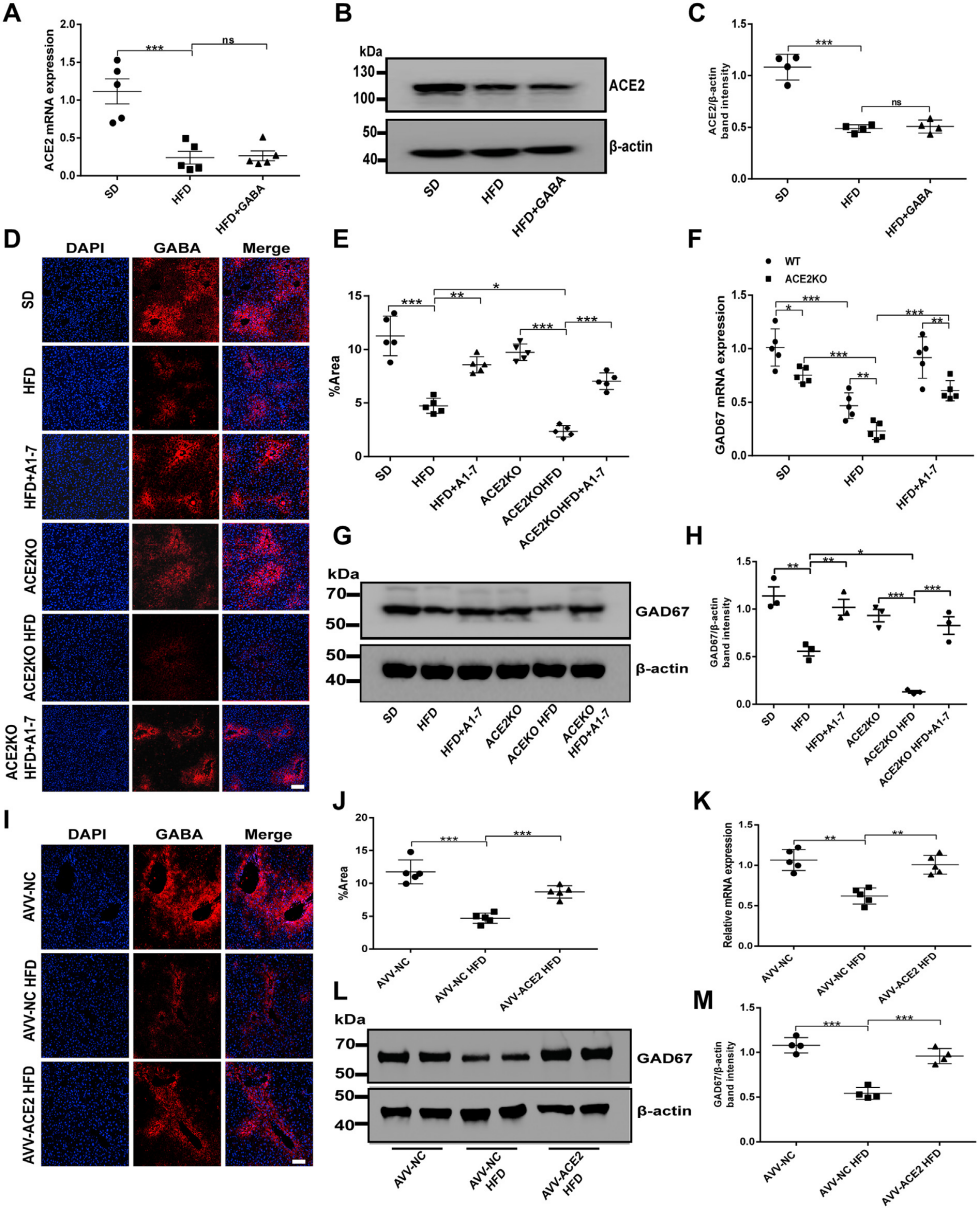
**ACE2/A1–7/MAS axis regulates GABAergic signaling expression in hepatocytes.***A*, the mRNA expression of hepatic ACE2 of WT mice treated with an HFD and/or GABA was measured by RT–quantitative PCR (qPCR). *B* and *C*, the protein expression of ACE2 in the liver of WT mice treated with HFD and/or GABA was measured by Western blot and densitometric quantification. *D* and *E*, representative immunostaining images of GABA (*red*) in the livers of WT or ACE2KO mice treated with HFD and/or A1–7 and the average absorbance of immunopositive cells. The scale bars represent 50 μm. *F*, the mRNA expression and (*G* and *H*) protein expression of GAD67 in the liver of WT or ACE2KO mice treated with HFD and/or A1–7 was measured. *I* and *J*, representative immunostaining images of GABA (*red*) in the livers of AVV-ACE2 mice treated with HFD and the average absorbance of immunopositive cells. The scale bars represent 50 μm. *K*, the mRNA expression and (*L* and *M*) protein expression of GAD67 in the liver of AVV-NC or AVV-ACE2 mice treated with HFD was measured. Values were expressed as means ± SEM (n = 5 or n = 6). ^∗^*p* < 0.05, ^∗∗^*p* < 0.01, ^∗∗∗^*p* < 0.001. A1–7, HFD mice were received with angiotensin 1 to 7 (576 μg/kg/day, i.p.); ACE2, angiotensin-converting enzyme 2; AVV, adeno-associated virus; AVV-ACE2 HFD, HFD feeding of AVV-mediated ACE2 overexpression mice; AVV-NC, mice were injected *via* tail vein with AVV-enhanced GFP; AVV-NC HFD, AVV-NC mice on HFD; GABA, HFD mice were received with gamma-aminobutyric acid (6 mg/ml in drinking water); GAD, glutamate decarboxylase; HFD, high-fat diet; NC, negative control; ns, no statistical difference; SD, standard diet.

**Supplemental Figure S2:**
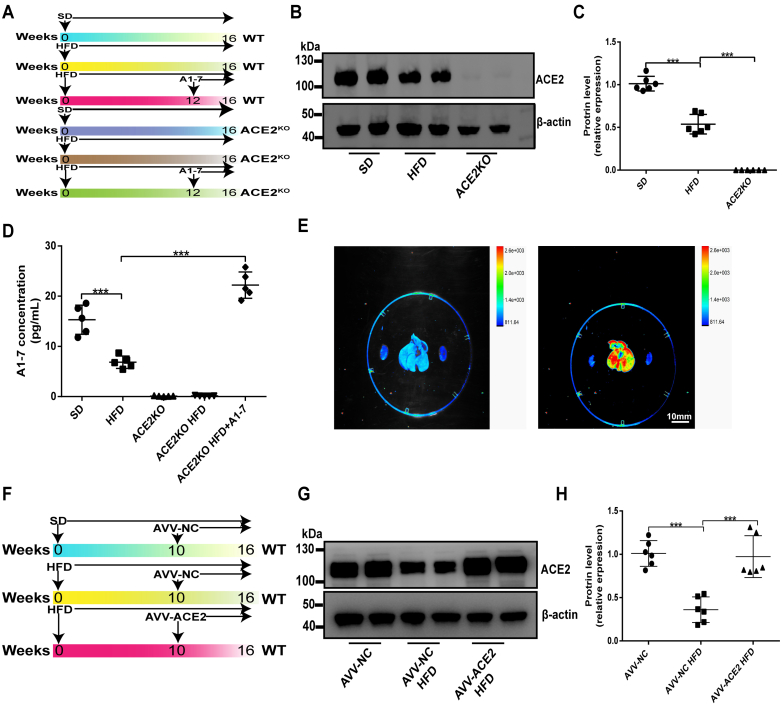


Studies have shown the regulatory effect of the ACE2/A1–7/MAS axis on the GABA signaling system in multiple organs (26, 27, 28). Therefore, we wanted to detect whether ACE2/A1–7/MAS axis has a regulatory effect on the local GABA signaling system in the liver under IR conditions. At first, the expression levels of ACE2 and GABA in the liver were detected in SD and HFD mice, respectively. Consistent with previous results, the ACE2 gene and protein expression levels in the liver of mice in the HFD group were significantly reduced in contrast to the SD group (Fig. 4, *A–C*). Similarly, mice with IR revealed an observably lessened expression of GABA and GAD67 in the liver (Fig. 4, *D–H*). Interestingly, we found that the reduction of GABA expression induced by high fat was reversed by the A1–7 intervention (Fig. 4, *D* and *E*), whereas GABA intervention has no significant effect on ACE2 expression (Fig. 4, *A–C*). Therefore, we hypothesized that ACE2/A1–7/MAS axis regulates the expression of GABAergic signaling in the liver. To examine the effects of ACE2 on GABA expression in the liver, we established ACE2KO mouse models (Fig. S2, *A–C*). The plasma concentrations of A1–7 were observed in SD mice and ACE2KO mice. A1–7 concentration was reduced in HFD mice, whereas no A1–7 concentrations were detected in ACE2KO mice with or without HFD. However, treatment with ACE2 significantly increased plasma A1–7 level (Fig. S2*D*). As seen in immunofluorescence staining of liver slices, GABA expression was reduced in ACE2KO SD mice compared with the SD group, and more significant losses were observed after HFD intervention, which was reversed by A1–7 treatment (Fig. 4, *D* and *E*). Comparably, the effect of ACE2 on hepatic GAD67 expression was also analyzed in ACE2KO HFD mice. Consistent with the changes in GABA expression, the mRNA expression level of GAD67 decreased significantly in ACE2KO HFD mice, whereas revealed a marked upregulation following exogenous A1–7 administration (Fig. 4*F*). Moreover, the protein level of GAD67 was consistent with the mRNA expression change (Fig. 4, *G* and *H*). Given the effect of ACE2 knockdown on hepatic GABA expression, we further confirmed the regulatory effect of ACE2 on GABA signaling by overexpressing hepatic ACE2. We found that enhanced GFP and ACE2 were successfully expressed in the liver of mice (Fig. S2, *E–H*). As with exogenous A1–7 intervention, hepatic ACE2 overexpression *via* adeno-associated virus (AVV) mediated significantly suppressed HFD-induced downregulation of hepatic GABA and GAD67 expression (Fig. 4, *I–M*). The aforementioned results show a close regulatory relationship between ACE2/A1–7/MAS axis and GABAergic signaling in the liver.

### Overexpression of ACE2 upregulates GABAergic signaling expression in palmitic acid–induced HepG2 cells

**Figure 5:**
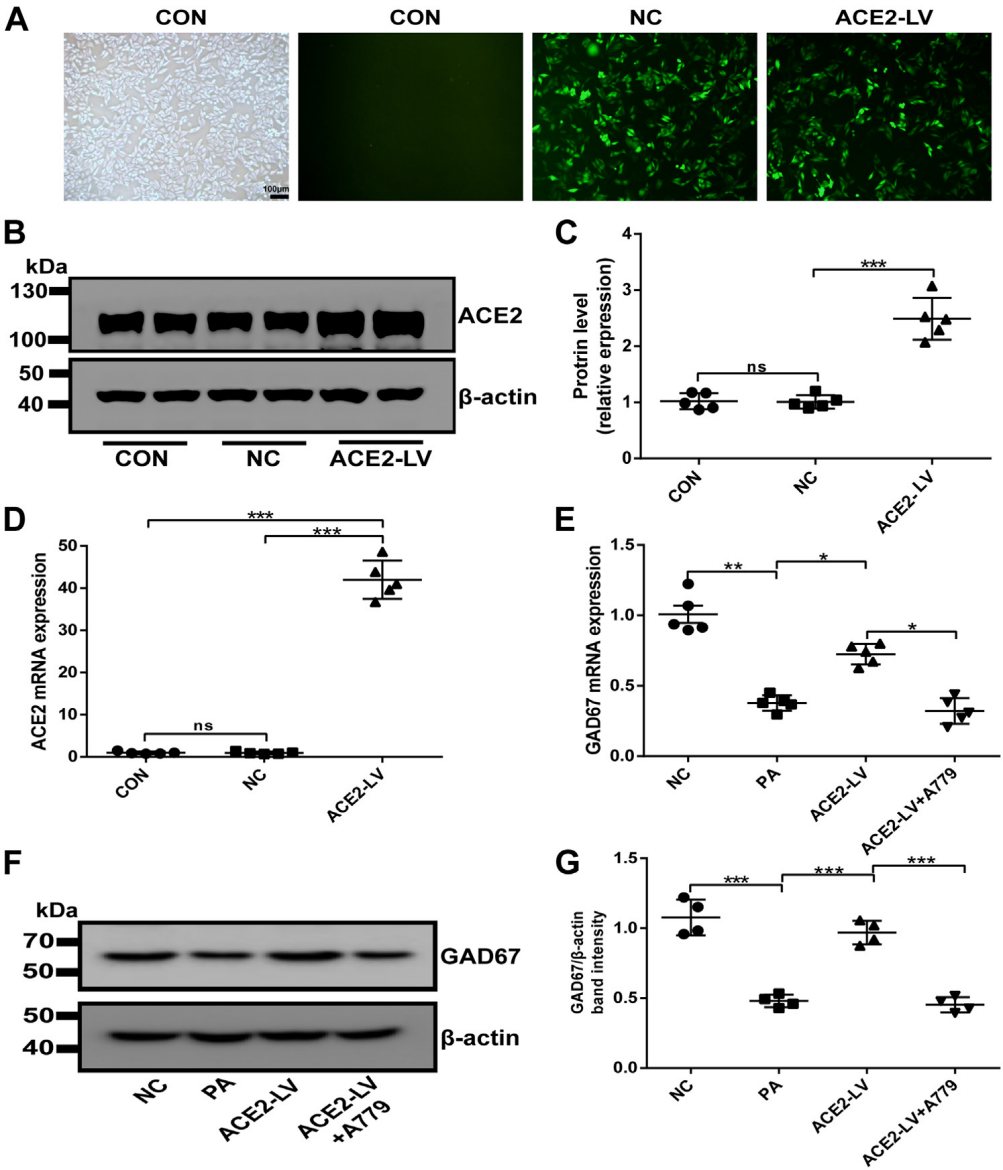
**The efficiency of the ACE2-GFP lentivirus infection in HepG2 cells 3 days after infection.***A*, HepG2 cells were transfected with recombinant lentivirus, and the viral titer of the recombinant lentivirus was observed under the inverted fluorescence microscope. The scale bars represent 100 μm. *B* and *C*, the protein expression of ACE2 in HepG2 cells was detected by Western blot and densitometric quantification. *D*, the mRNA expression of ACE2 in HepG2 cells was detected by RT–quantitative PCR analysis. *E*, the mRNA expression and (*F* and *G*) the protein expression of GAD67 in LV-ACE2-treated HepG2 cells. Values were expressed as means ± SEM (n = 4 or 5). ^∗^*p* < 0.05, ^∗∗^*p* < 0.01, ^∗∗∗^*p* < 0.001. ACE2, angiotensin-converting enzyme 2; ACE2-LV, ACE2 overexpression group infected with ACE2-GFP lentiviral vectors were treated with PA; ACE2-LV + A779, ACE2 overexpression group was treated with PA and A779 (10 μmol/l); CON, control group without intervention; GAD67, GAD, glutamate decarboxylase; LV, lentivirus vector; NC, negative control group infected with GFP lentiviral vector; ns, no statistical difference; PA, palmitic acid.

Our results confirmed that A1–7 intervention could regulate the hepatic GABAergic signaling expression *in vivo*. In order to eliminate environmental effects on the liver, the experiments were performed to directly investigate the regulatory effect of ACE2 on GABA *in vitro*. ACE2-overexpressed HepG2 cells were constructed *via* ACE2 lentivirus transfection. After lentivirus infection, most HepG2 cells expressed GFP (Fig. 5*A*), and PCR and Western results indicated successful lentivirus packaging and cell infection (Fig. 5, *B–D*). As indicated in Figure 5, *E–G*, palmitic acid (PA)–induced HepG2 cells markedly downregulated the gene and protein expression of GAD67. In contrast, the overexpression of ACE2 significantly upregulated the expression of GDA67 mRNA and protein compared with the PA group, which was consistent with the results *in vivo*. However, the effect of ACE2 overexpression on GAD67 in HepG2 cells was attenuated by A779 (MAS-specific inhibitor) (Fig. 5, *E–G*). In summary, these results reveal that ACE2/A1–7/MAS axis modulates hepatic GABAergic signaling expression under IR *in vitro*, which is consistent with *in vivo* results.

### The blockade of the GABAergic signaling attenuates the ACE2-mediated benefit in PA-induced HepG2 cells

**Figure 6:**
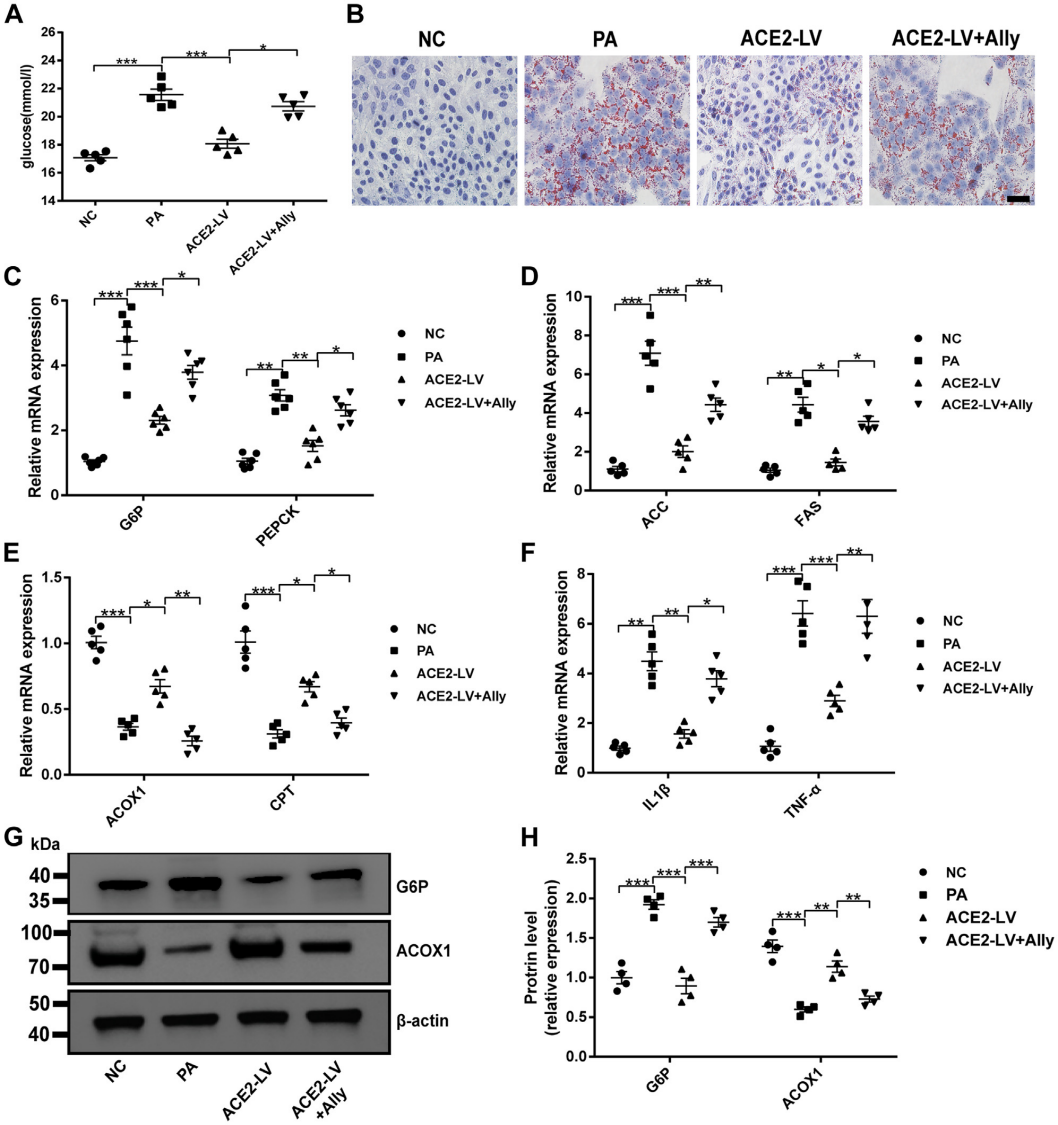
**A blockage of the GABAergic signal attenuates ACE2-mediated benefits on HepG2 cells.***A*, glucose concentration in cell supernatant. *B*, Oil *Red* O staining of HepG2 cells. The scale bars represent 100 μm. *C*–*F*, relative mRNA expression of gluconeogenesis-related genes G6P and PEPCK (*C*), fatty acid synthesis–related genes ACC and FAS (*D*), fatty acid oxidation–related genes ACOX1 and CPT (*E*), and inflammation-related genes IL-1β and TNF-α (*F*) in HepG2 cells was measured by quantitative PCR. *G* and *H*, the protein expression of G6P and ACOX1 in HepG2 cells. Values were expressed as means ± SEM (n = 4 or 5). ^∗^*p* < 0.05, ^∗∗^*p* < 0.01, ^∗∗∗^*p* <  0.001. ACC, acetyl-CoA carboxylase; ACE2, angiotensin-converting enzyme 2; ACE2-LV, ACE2 overexpression group was treated with PA; ACE2-LV + ally, ACE2 overexpression group was treated with PA and allylglycine (5 mmol/l); ACOX1, acyl-CoA oxidase 1; CPT, carnitine palmityl transferase; FAS, fatty acid synthase; GABA, gamma-aminobutyric acid; IL-1β, interleukin 1β; NC, negative control group infected with GFP lentiviral vector; PA, palmitic acid; TNF-α, tumor necrosis factor alpha.

The aforementioned results indicate that the ACE2/A1–7/MAS axis regulates the expression of GABAergic signaling in the liver under IR *in vivo* and *in vitro*. Our findings confirm that ACE2/A1–7/MAS axis and GABA can improve hepatic IR. However, it remains unclear whether the GABAergic signal regulation mediated by the ACE2/A1–7/MAS axis is functionally related to its beneficial effects on the liver. To further clarify whether ACE2-mediated protection of hepatic IR is related to GABAergic signaling, the effect of pharmacological inhibition of GABA signaling by allylglycine, a specific inhibitor of GAD, on ACE2-mediated IR in hepatocytes was investigated. In comparison with the CON group, PA reduced glucose uptake and utilization by HepG2 cells, and ACE2 overexpression significantly increased glucose consumption. However, the addition of allylglycine almost blocked the effect of ACE2 (Fig. 6*A*). Oil Red O staining was used to observe lipid accumulation in HepG2 cells. Intracellular lipid drop content strikingly increased after PA treatment, and ACE2 overexpression inhibited PA-induced lipid accumulation (Fig. 6*B*). Importantly. ACE2 overexpression–mediated improvement of fat deposition was largely eliminated by adding allylglycine to inhibit GABAergic signaling (Fig. 6*B*). Analogously, glucolipid metabolism and inflammation-related gene expression were investigated. The expression of gluconeogenesis-related genes (G6P and PEPCK) and lipid synthesis–related genes (ACC and FAS) was significantly upregulated after PA treatment, whereas the mRNA expression of ACOX-1 and CPT-1 was downregulated in comparison with the negative control (NC) group, which was prevented by ACE2 overexpression in HepG2 cells as expected (Fig. 6, *C–E*). In addition, allylglycine noticeably weakened the inhibition of ACE2 on G6P, PEPCK, ACC, and FAS mRNA expression and blocked the promotion effect of ACE2 on ACOX-1 and CPT-1 expression (Fig. 6, *C–E*). Consistent with the mRNA expression change in PA-treated HepG2 cells, the protein levels of G6P had significantly increased as seen by Western blot, whereas ACOX protein level was decreased, and exogenous A1–7 treatment reversed these changes (Fig. 6, *G–H*). We further measured two important inflammatory cytokines in the current study. As shown in Figure 6*F*, inflammation was induced by PA, mainly manifested as increased IL-1β and TNF-α compared with the NC group, whereas ACE2 overexpression in HepG2 cells weakened the PA-induced inflammatory response to a large extent. Importantly, ACE2 failed to prevent this effect when cotreated with allylglycine (Fig. 6*F*). These results suggest that the beneficial effect of ACE2 on hepatocyte IR is mediated at least in part by regulating GABAergic signaling.

### ACE2 regulates hepatic GABAergic signaling to improve hepatic IR effects at least partially mediated by PI3K/AKT signaling

**Figure 7:**
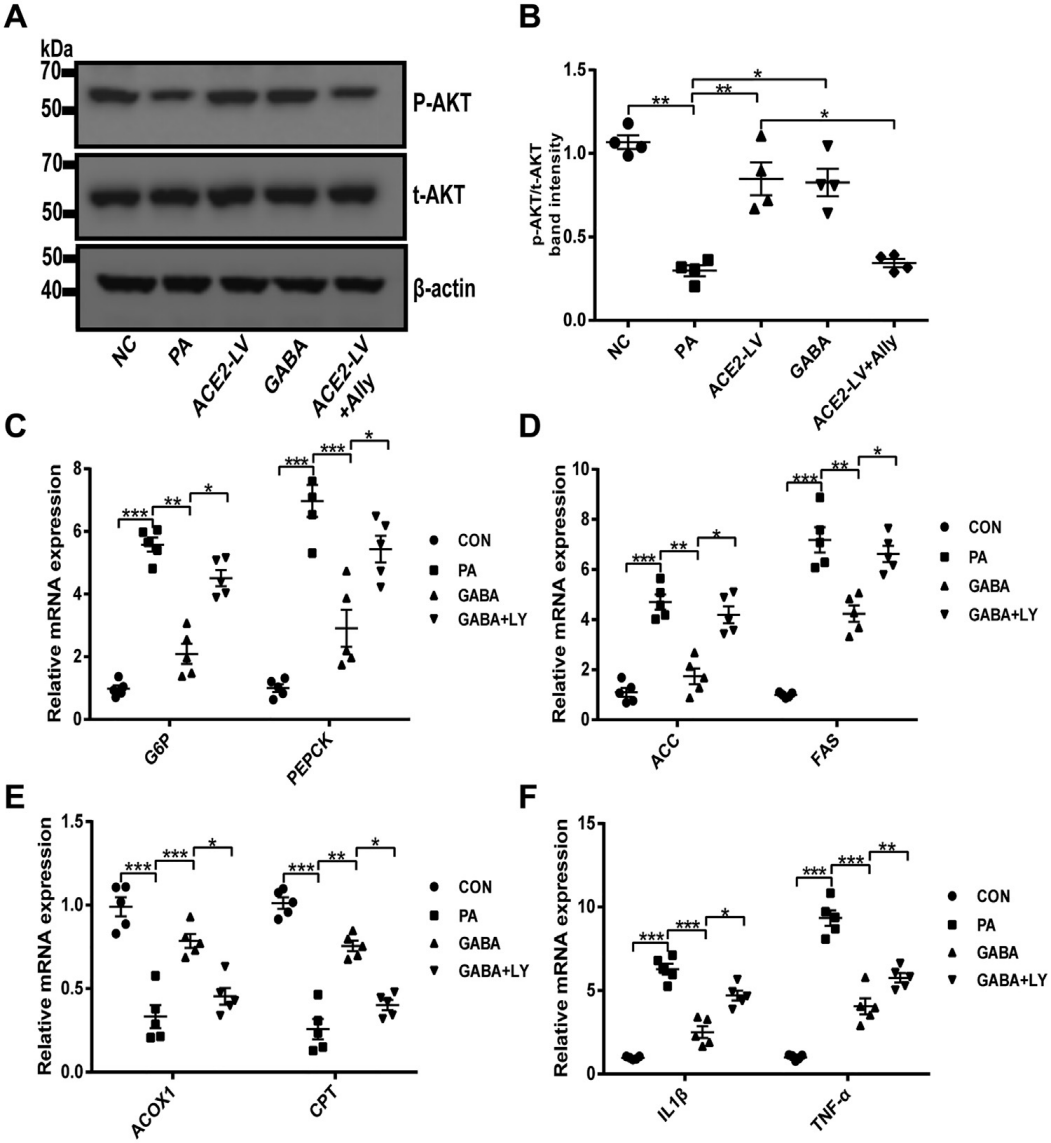
**ACE2 regulates PI3K/AKT signaling by upregulating GABAergic signaling to improve hepatic insulin resistance.***A* and *B*, Western blotting of AKT phosphorylation in HepG2 cells and densitometric quantification after insulin stimulation. *C*–*F*, relative mRNA expression of gluconeogenesis-related genes G6P and PEPCK (*C*), fatty acid synthesis–related genes ACC and FAS (*D*), fatty acid oxidation–related genes ACOX1 and CPT (*E*), and inflammation-related genes IL-1β and TNF-α (*F*) in HepG2 cells was measured by quantitative PCR. Values were expressed as means ± SEM (n = 4 or 5). ^∗^*p* < 0.05, ^∗∗^*p* < 0.01, ^∗∗∗^*p* < 0.001. ACC, acetyl-CoA carboxylase; ACE2, angiotensin-converting enzyme 2; ACE2-LV, ACE2 overexpression group was treated with PA; ACE2-LV + ally, ACE2 overexpression group was treated with PA and allylglycine (5 mmol/l); ACOX1, acyl-CoA oxidase 1; CPT, carnitine palmityl transferase; FAS, fatty acid synthase; GABA, gamma-aminobutyric acid; GABA + LY, GABA group was treated with PA and LY294002 (20 μmol/l); IL-1β, interleukin 1β; NC, negative control group was infected with GFP lentiviral vector; PA, palmitic acid; TNF-α, tumor necrosis factor alpha.

It has been reported that PI3K/AKT pathway is one of the crucial downstream signaling pathways of GABAergic signaling (31), and PI3K/AKT pathway is also closely related to the regulation of glycolipid metabolism and inflammation (32). Therefore, we further investigated whether GABA can improve hepatic IR through PI3K/AKT pathway. Correspondingly, Akt activity was inspected in HepG2 cells by Western blot. As shown in Figure 7, *A* and *B*, the protein level of p-AKT was downregulated after PA treatment in contrast to the NC group, whereas ACE2 overexpression in HepG2 cells or GABA intervention significantly increased the p-AKT level. The effect of ACE2-mediated upregulation of AKT phosphorylation was almost blocked by allylglycine. It has been preliminarily confirmed that AKT mediates the protective effect of ACE2 on hepatic IR as a downstream signaling pathway of GABA. To further test this hypothesis, the effect of pharmacological inhibition of PI3K/AKT signaling by LY294002 (a specific inhibitor of PI3K) was investigated. The results showed that GABA significantly inhibited PA-induced upregulation of gluconeogenesis-related genes (G6P and PEPCK), lipid synthesis–related genes (ACC and FAS), and inflammatory factors (IL-1β and TNF-α) (Fig. 7, *C*, *D* and *F*) as well as promoted the expression of fatty acid β oxidation–related genes (ACOX-1 and CPT) (Fig. 7*E*), whereas LY294002 supply significantly weakened the effect of GABA (Fig. 7, *C–F*). The aforementioned results suggested that the effect of ACE2 on improving hepatic IR *via* GABAergic signaling is mediated at least in part by PI3K/AKT signaling.

## Discussion

It has been found that ACE2/A1–7/MAS axis and GABAergic signaling are both expressed in the liver and play an essential protective role in the liver. Still, their relationship in the liver remains unclear. A previous study confirmed that the ACE2/A1–7/MAS axis in islets improves β-cell dysfunction by promoting the GABAergic signaling system. Interestingly, in the current study, we identified ACE2/A1–7/MAS axis as an essential protective mechanism in the pathogenesis of hepatic IR by regulating the expression of hepatic GABAergic signaling. We revealed that ACE2/A1–7/MAS axis effectively promoted the expression of the GABAergic signaling system under metabolic stress, thereby regulating glucose and lipid metabolism, weakening the inflammatory response, and ultimately lessening hepatic IR.

An intact ACE2 axis exists in the liver (33), but its function in hepatic IR has not been analyzed in depth. As we all know, the liver plays a crucial role in metabolic homeostasis. It is the prominent place for the metabolism, synthesis, and redistribution of carbohydrates, glucose, and lipids and the development of IR (34, 35). Reducing body weight is considered to be an effective means of improving insulin sensitivity. Indeed, our data suggested that A1–7 treatment greatly regulated the increase in body weight and fat accumulation without affecting the food intake, which might be related to the regulation of adipogenesis and adipolysis-related genes. HFD-fed mice showed persistent hyperglycemia symptoms suggesting the mice developed insulin tolerance and impaired glucose tolerance. Interestingly, A1–7 treatment could markedly improve hepatic glucose intolerance. In addition, we found that A1–7 treatment had a practical impact on the serum lipid levels and improved liver histomorphological abnormalities by H&E staining and Oil Red O staining. These results are consistent with the conclusions obtained in HFD-fed mice treated with angiotensin receptor antagonists (angiotensin receptor blockers), which alleviate hepatic steatosis by activating ACE2/A1–7/MAS axis (36). IR involves regulating multiple genes at the transcriptional and translational levels. ACE2 KO increased the expression of FAS and ACC, which are critical enzymes for fatty acid synthesis in the liver, whereas decreasing the gene expression of fatty acid β oxidation–related enzymes. Overexpression of ACE2 in the liver or intervention with exogenous A1–7 significantly inhibited lipogenesis and enhanced fatty acid β oxidation as well as downregulated the expression of PEPCK and G6P in the liver (25, 37, 38). Consistent with these data, our findings confirm the benefits of A1–7 on hepatic IR. In addition, it has been demonstrated that increased oxidative stress and local proinflammatory states can influence IR, making it an intervention target for ameliorating type 2 diabetes (39, 40). Macrophages are an essential part of the mononuclear phagocyte system and have a wide range of functions in the body. Macrophages in the liver are mainly distributed around the hepatic sinusoids. In the state of obesity-induced IR, the increase of inflammatory cytokines in serum and tissues can secrete various inflammatory chemokines to activate M1-type macrophages, increase the infiltration of macrophages in the liver, and secrete more inflammatory factors, thus aggravating hepatic IR (30, 41). Studies have shown that A1–7 can significantly inhibit the secretion of proinflammatory cytokines by lipopolysaccharide-induced activated macrophages and reduce the body's inflammatory response (42). The current research found that macrophage markers and proinflammatory cytokines increased in liver tissues under IR. However, treatment with A1–7 significantly suppressed the infiltration of macrophages in the liver and then caused less release of IL-1β and TNF-α. These results further confirmed the potential anti-inflammatory effect of ACE2/A1–7/MAS on HFD-fed mice.

Several recent studies have demonstrated the therapeutic effects of GABA on hyperglycemia. Pancreatic β cells can synthesize large amounts of GABA, which plays an important protective role. The content of GABA and GAD in islets is significantly reduced in diabetes (43, 44, 45). Our previous studies found that GABA and GAD levels in islet β-cells also decreased significantly under IR (28). Interestingly, the liver also has a complete GABAergic signaling system. Wang *et al.* (13) had shown that GABA and GABA–AR expression levels were significantly downregulated in the liver of rats with acute liver injury, whereas the expression level of GAD67 was upregulated. This study believed that the upregulated expression of GAD67 in the liver was a compensatory effect of the body's response to liver injury. However, there are few studies on the hepatic local GABA signal changes under IR. Previous studies have shown that ACE2/A1–7/MAS axis and GABAergic system are expressed in the liver and play an important role, but the relationship between these two systems in the liver remains unclear. It has been demonstrated that various interactions between the RAS and GABAergic signaling system *in vivo* are related to blood pressure and mood (26, 27). Some neurotrophic factors can also activate downstream Ras/ERK signaling pathway through ACE2/A1–7/MAS axis, and phosphorylation of CREB promotes GAD transcription and upregulates GABA expression (46). We also confirmed in previous studies that upregulation of the ACE2/A1–7/MAS axis by exogenous A1–7 intervention could improve GABA expression in pancreatic islets and improve β-cell function in rats with metabolic stress model (28). In the present study, we found that the expression of ACE2 and GABA decreased in the liver in IR models. Furthermore, the levels of GAD67 and GABA were downregulated more significantly in ACE2KO mice specially fed with an HFD, whereas they were reversed by exogenous A1–7 treatment. However, exogenous GABA did not upregulate ACE2 expression. In addition, we further confirmed the regulatory effect of ACE2 on GABA through AVV-mediated hepatic ACE2 overexpression. Consistent with these *in vivo*, overexpression of ACE2 upregulated GAD67 level in PA-treated HepG2 cells. To confirm whether the regulation of the GABAergic signaling system by ACE2 is related to its effect on hepatic IR, we employed the GAD67 in/hibitor allylglycine in PA-induced HepG2 cells. We found that the regulatory effect of ACE2 on glucose and lipid metabolism and the inhibitory effect on inflammation were significantly weakened by allylglycine. Therefore, this evidence demonstrated that the hepatic ACE2/A1–7/MAS axis could play a role in improving IR by regulating the hepatic GABAergic signaling system.

This study also confirmed the direct protective effect of the GABA signaling system on liver IR through upregulation of the hepatic GABAergic signaling system. It has been demonstrated that glycogen synthase 1/2 is primarily in charge of hepatic glycogen synthesis, improving IR by promoting hepatic glycogen production. Shang *et al.* (14) demonstrated that GABA dietary intervention improved IR-induced hyperinsulinemia and reduced blood glucose levels by promoting glycogen synthase 1/2 gene expression. In addition, GABA dietary intervention can also promote the uptake and utilization of glucose in the liver and muscle by inhibiting the expression of gluconeogenesis-related genes and upregulating the expression of GLUT2 (14, 47) and promoting fatty acid β oxidation and reducing liver fat deposition by enhancing the activity of peroxisome proliferators–activated receptor transcription factors (48). Our results are consistent with the aforementioned studies. In this study, the intervention of GABA with IR model mice and in PA-induced HepG2 cells demonstrated that GABA could significantly improve the balance of liver glucose and lipid metabolism and enhance insulin sensitivity, as evidenced by inhibiting the expression of lipogenesis and gluconeogenesis-related genes and proteins in the liver and promoting the expression of fatty acid β oxidation–related genes. Tian *et al.* (49) found that oral GABA intervention significantly reduced the infiltration of macrophages induced by high fat in adipose tissue. However, there are few studies on the effect of GABA on hepatic macrophages under IR. In the current research, we found that GABA intervention can significantly reduce the infiltration of macrophages in the liver and then cause less expression of IL-1β and TNF-α. These results further confirmed the potential improvement effects of GABA in HFD-induced IR in mice.

As mentioned in previous studies, PI3K/AKT signaling pathways are a canonical pathway downstream of insulin, regulating glycogen synthesis, gluconeogenesis, and glucose transport and utilization. Meanwhile, the activation of this pathway is also closely related to lipid metabolism (50, 51). Studies have found that in the state of IR, the insulin signaling pathway is damaged, and PI3K and AKT phosphorylation is significantly inhibited, which stimulates liver fat deposition and gluconeogenesis, leading to liver glucose and lipid metabolism disorder (32). Interestingly, PI3K/AKT signaling pathway is also one of the critical cellular signaling pathways downstream of the GABAergic signaling system. GABA plays a therapeutic effect on schizophrenia by increasing AKT phosphorylation levels and reducing GSK-3 expression (31). Purwana *et al.* (45) found that GABA can protect pancreatic β cells and promote the regeneration of β cells by activating the PI3K/AKT signaling pathway, thus playing a role in the treatment of diabetes. As shown in the aforementioned description, GABA significantly increased phosphorylated AKT levels in the liver of HFD-fed mice and PA-treated HepG2 cells and improved IR, which was partially reversed by LY294002 (an Akt inhibitor). These results revealed that GABA might ameliorate hepatic IR *via* activating the PI3K/AKT signaling pathway.

In conclusion, our data suggest that the liver GABAergic signaling system may be an essential mediator in the protective effect of the ACE2/A1–7/MAS axis on the liver, which can improve lipid accumulation and gluconeogenesis, prevent inflammation by upregulating liver PI3K/AKT signaling pathway, and thus mediating the protective effect of ACE2/A1–7/MAS axis on hepatic IR.

## Experimental procedures

### Chemicals

GABA was purchased from Sigma–Aldrich, and A1–7 and A779 were purchased from Bachem. Allylglycine (a specific GAD67 inhibitor) and LY294002 (a PI3K inhibitor) were obtained from MedChem Express. Antibodies against β-actin, AKT, p-AKT (Ser473), GAD67, GABA, G6P, and ACOX1 were purchased from Cell Signaling Technologies. Antibodies against ACE2 and F4/80 were purchased from Abcam. The secondary antibodies of goat anti-rabbit, goat antimouse, and rabbit antisheep were purchased from Abcam. The TG and TC assay kits were obtained from Bio-Rad. Serum levels of insulin, A1–7, IL-1β, and TNF-α were measured using ELISA kits (MU30432, MU30979, MU30030, and MU30369; Bio-Swamp).

### Animals and treatments

#### A1–7 and GABA treatment models

Male C57BL/6J mice (6 weeks of age) were used in this study, obtained from Beijing HFK Biotechnology Co, Ltd. Mice were randomly divided into four groups (n = 5 per group): SD group, HFD group, A1–7 group, and GABA group. The SD group was fed regular chow, whereas the remaining mice were fed an HFD (Research Diets; catalog no.: D12492) containing 60% calories for 3 months to induce IR. Mice in the A1–7 group were treated with A1–7 (576 μg/kg/day, i.p.) (18) and in the GABA group was treated with GABA (6 mg/ml in drinking water) (28), whereas in the SD group and HFD group, mice were intraperitoneally injected with the equivalent volume of sterile saline for 4 weeks.

#### ACE2KO mouse mode

Male ACE2KO mice (6 weeks of age, on a C57BL/6J background) and their male WT littermates were obtained from the Institute of Laboratory Animal Science, Chinese Academy of Medical Sciences, and randomly divided into six groups (n = 5 per group): SD group, HFD group, A1–7 group, ACE2KO group, ACE2KO HFD group, and ACE2KO HFD + A1–7 group. Mice were fed an SD or an HFD for 12 weeks. Mice in the A1–7 group and ACE2KO HFD + A1–7 group were treated with A1–7 (576 μg/kg/day, i.p.), whereas the other four groups were intraperitoneally injected with the equivalent volume of sterile saline for 4 weeks.

#### AVV-based ACE2 overexpression mouse model

Male C57BL/6J mice (6 weeks of age) from Beijing HFK Biotechnology Co, Ltd were used in this study. Mice were randomly divided into five groups (n = 5 per group): AVV-NC group, AVV-NC HFD group, and AVV-ACE2 HFD group. The AVV-NC group was fed with regular chow, whereas the remaining mice were fed an HFD (Research Diets; catalog no.: D12492) containing 60% calories for 3 months to induce IR. AVVs are diluted to 3 × 10^9^/μl in injection buffer. Mice were fed HFD for 10 weeks and then were injected *via* tail vein with 3 × 10^11^ AVV-enhanced GFP/mice or 3 × 10^11^ AVV-ACE2/mice (serotype: AVV9). The expression of ACE2 was assessed using RT–PCR or Western blot analysis.

Mice were maintained under controlled standards with chow and water in light and temperature. All experiments were performed according to procedures approved by the Animal Research Committee of Tongji Medical College, Huazhong University of Science and Technology, Hubei Province, China.

#### Blood glucose levels, intraperitoneal insulin tolerance tests, and intraperitoneal glucose tolerance tests

Tail fasting blood glucose was measured using a blood glucose meter (LifeScan) after mice fasted for 12 h. The intraperitoneal glucose tolerance test was performed by injecting glucose (2 g/kg body weight, i.p.) to mice, which were fasted for 12 h. Blood was collected from the tip of the tail vein and measured at 0, 30, 60, 90, and 120 min. The intraperitoneal insulin tolerance test was performed by injecting insulin (0.75 U/kg body weight, i.p.) to mice, which were fasted for 12 h. Blood was collected from the tail vein's tip and measured at 0, 15, 30, 60, and 90 min. The area under the curve was calculated by GraphPad Prism 7.00 software (GraphPad Software, Inc).

#### CT imaging of liver

The mice were anesthetized with 2% isoflurane and placed on a scanning bed; Gem CT (Discovery CT 750 HD; GE) was used for energy spectrum scanning. The head and limbs were fixed with cellophane tape in a supine position. First, the mutually vertical positioning phase was obtained, and the scanning range was from the top of the diaphragm to the lower edge of the liver. The 65 keV single-photon image was automatically generated using energy spectrum scanning mode (gemstone spectral imaging) and a standard reconstruction algorithm. The 65 keV single-photon CT image was loaded into gemstone spectral imaging general postprocessing software, and the layer with a relatively uniform density of liver parenchyma was selected. The liver CT values of 65 keV single-energy images were measured by clicking the material analysis option, and the energy spectrum curve was generated.

#### Histological analysis and detection of lipid content

After the mice were sacrificed, the liver tissues were fixed in 4% paraformaldehyde (Sigma–Aldrich), embedded in paraffin blocks, and sequentially sliced into 5 μm sections. Liver slices were stained with H&E and Oil Red O to evaluate hepatic histology and steatosis changes. The content of TG and TC in the serum and liver tissues of mice was further detected using an enzyme standard from a TG and TC diagnostic kit (Nanjing Jiancheng).

#### Immunofluorescence staining

The paraffin sections of liver tissues were deparaffinized with xylene and then dehydrated in 100, 95, 85, and 70% ethanol. The sections were incubated with primary antibodies overnight at 4 °C (rabbit anti-GABA 1:100 dilution, rabbit anti-F4/80 1:100 dilution). After washing three times in PBS for 10 min, the sections were incubated with an anti-rabbit FITC-conjugated secondary antibody (1:100 dilution) for 1 h, and nuclei were stained with 4′,6-diamidino-2-phenylindole (18). The sections were observed by light microscopy and captured using ImageJ software (National Institutes of Health).

#### Construction of stable ACE2-expressing HepG2 cells by lentivirus transfection

A customized ACE2-overexpression lentivirus vector or LV-NC was obtained from Genechem. HepG2 cells were transfected with the LV-ACE2 following the manufacturer’s protocols and selected using puromycin (2 μg/ml; Sigma–Aldrich). The surviving cells were cultured into multiple monoclonal cell lines and assessed for the expression of ACE2 using RT–PCR or Western blot analysis.

#### Cell treatments

The HepG2 cell line was purchased from American Type Culture Collection and maintained in Dulbecco's modified Eagle's medium (DMEM) with 10% fetal bovine serum at 37 °C in an atmosphere of 95% humidified air and 5% CO_2_. We created an IR model *in vitro* by PA. The experiments on HepG2 cells were divided into two parts: (1) LV-NC and LV-ACE2 HepG2 cells were treated with or without PA (0.25 mmol/l) and/or cocultured with A779 (10 μmol/l), GABA (100 μmol/l), and allylglycine (5 mmol/l) for 24 h, respectively; (2) HepG2 cells were treated with or without PA (0.25 mmol/l) in the presence or the absence of GABA (100 μmol/l) or PI3K inhibitor LY294002 (20 μmol/l) for 24 h (19, 28).

### Real-time quantitative RT–PCR

Total RNA from hepatic tissue and cultured HepG2 cells was extracted using TRIzol reagent (Takara Shuzo Co, Ltd), and the complementary DNA was synthesized using a PrimeScript RT reagent kit (Takara Biotechnology Co Ltd). Then quantitative PCR was performed in a LightCycler480-PCR system (Roche Diagnostics). The relative transcript levels of the target gene were normalized to GAPDH and calculated by the 2^−ΔΔCT^ method. The primer sequences are listed in Table 1.

**Table 1:** List of primers used for real-time PCR using SYBR-Green

Gene	Forward sequence (5′ to 3′)	Reverse sequence (5′ to 3′)
G6P(m)	AGACTCCCAGGACTGGTTCA	GTCCAGGACCCACCAATACG
PEPCK(m)	ACACCAATGGGGGTTTTGGT	AAAGGTAAGGAAGGGCGGTG
ACC(m)	ATGGGCGGAATGGTCTCTTTC	TGGGGACCTTGTCTTCATCAT
FAS(m)	GGAGGTGGTGATAGCCGGTAT	TGGGTAATCCATAGAGCCCAG
ACOX-1(m)	AGGTTGTCATCGCTTTGG	GTGATTAACTCTGGATTGAAG
CPT-1(m)	ACTCCGCTCGCTCATTCCG	CACACCCACCACCACGATAA
IL-1β(m)	TGCCACCTTTTGACAGTGATG	AAGGTCCACGGGAAAGACAC
TNF-α(m)	GACGTGGAACTGGCAGAAGAG	TTGGTGGTTTGTGAGTGTGAG
ACE2(m)	ACACTCTGGGAATGAGGACAC	ACACTCTGGGAATGAGGAC
GAD67(m)	TCCGGCTTTTGGTCCTTCG	ATGCCGCCCGTGAACTTTT
G6P(h)	CACTTCCGTGCCCCTGATAA	GTAGTATACACCTGCTGTGCCC
PEPCK(h)	AGTGATGGTGGCGTGTACTG	CTGGGACTGGAAACTGCAAAC
ACC(h)	CATGCGGTCTATCCGTAGGTG	GTGTGACCATGACAACGAATCT
FAS(h)	ACAGCGGGGAATGGGTACT	GACTGGTACAACGAGCGGAT
ACOX-1(h)	GCTGGAGCTGCGGATTTAGA	AGCTTTTCTCGGGAAAGGAGGC
CPT-1(h)	TTTGGACCGGTTGCTGATGA	TTTGGACCGGTTGCTGATGA
IL-1β(h)	TGAGCTCGCCAGTGAAATGAT	TCCATGGCCACAACAACTGA
TNF-α(h)	CCTCTCTCTAATCAGCCCTCTG	GAGGACCTGGGAGTAGATGAG
ACE2(h)	CATTGGAGCAAGTGTTTGGATCTT	GAGCTAATGCATGCCATTCTCA
GAD67(h)	CCTCAACTATGTCCGCAAGAC	TGTGCGAACCCCATACTTCAA
GAPDH(h)	AGCCTCGTCCCGTAGACAAAAT	CCGTGAGTGGAGTCATACTGGA

### Western blotting

Protein from cultured HepG2 cells was extracted in radioimmunoprecipitation assay lysis buffer (Beyotime) with protease and phosphatase inhibitors. Homogenates were centrifuged at 12,000 rpm for 10 min at 4 °C. The supernatants were collected to determine protein concentration using the bicinchoninic acid assay (Beyotime). Protein samples were separated by 10% SDS-PAGE and transferred to a polyvinylidene difluoride membrane. The membrane was blocked with 5% nonfat milk and incubated with primary antibodies against ACE2, GAD67, G6P, ACOX1, AKT, and p-AKT (Ser473) overnight at 4 °C. Membranes were washed three times with Tris-buffered saline with Tween-20 for 10 min and incubated in secondary antibodies (diluted 1:3000) for 1 h at room temperature. Finally, the membranes were washed three times in Tris-buffered saline with Tween-20 for 5 min and visualized with an enhanced chemiluminescence reagent (Millipore) and quantified using ImageJ software (National Institutes of Health).

### Glucose uptake

The cell supernatant was collected and measured at 550 or 570 nm using an enzyme standard from a glucose diagnostic kit after incubating with 100 nmol/l insulin for 20 min at 37 °C. Glucose content was calculated from a standard curve based on the absorbance of each sample. The glucose content of the experimental groups and the original DMEM were measured. Glucose uptake was quantified by the difference value between the original DMEM and the medium supernatant of the experimental group medium.

### Statistical analysis

GraphPad Prism 7.00 software was used to analyze and process the data. Differences in numeric parameters between two groups were assessed with an unpaired two-tailed *t* test, and a one-way ANOVA was used to compare multiple groups. All data were expressed as the mean ± SEM, and *p* < 0.05 was considered statistically significant.

## Data availability

All data are contained within the article.

## Supporting information

This article contains supporting information.

## Conflict of interest

The authors declare that they have no conflicts of interest with the contents of this article.

## Supplementary data

Supplemental Figures S1–S2 Captions
