# Supplementary material for: Angiotensin-converting enzyme 2 improves hepatic insulin resistance by regulating GABAergic signaling in the liver
Source: J Biol Chem. 2022 Oct 18;298(12):102603. doi: 10.1016/j.jbc.2022.102603 (PMC9668738; doi:10.1016/j.jbc.2022.102603)
Supplement: Supplemental Figures S1–S2 Captions [file mmc1.docx]

**Supplementary Figure 1** Metabolic Phenotype of mice upon HFD Challenge, related to Figure 1. (A) Epididymal fat weight. (B) Average food intake. (C) Plasma insulin levels at the indicated times during IPGTT assays. Values were expressed as means ± SEM (n =5 or n=6). ^*^P < 0.05, ^**^P < 0.01, ^***^P < 0.001. SD, standard diets; HFD, high-fat diets; A1–7, HFD mice received angiotensin 1–7 (576μg/kg/day, i.p.); GABA, HFD mice received gamma-aminobutyric acid (6mg/ml in drinking water).

**Supplementary Figure 2** Construction of ACE2KO and AVV-ACE2 mouse models, related to Figure 4. (A)The experiment schedule of the ACE2KO mouse model. (B, C) The protein expression of ACE2 in the liver of WT or ACE2KO mice. (D) Serum A1-7 levels. (E) Liver imaging of mice 14 days after tail vein injection of AAV-EGFP. (F) The experiment schedule of the AVV-ACE2 mouse model. (G, H) The protein expression of ACE2 in the liver of AVV-NC or AVV-ACE2. Values were expressed as means ± SEM (n =5 or n=6). ^*^P < 0.05, ^**^P < 0.01, ^***^P < 0.001. SD, standard diets; HFD, high-fat diets; ACE2KO, ACE2 knockout mice; AVV-NC, mice were injected via tail vein with AAV-eGFP; AVV-NC HFD, AVV-NC mice on high-fat diets; AVV-ACE2 HFD, HFD feeding of AVV-mediated ACE2 overexpression mice.
